# APOE Genotype Modulates Tau Propagation in A Novel Humanized Mouse Model of Early‐Stage Alzheimer's Disease

**DOI:** 10.1002/alz70855_106221

**Published:** 2025-12-24

**Authors:** Hirra A Arain, Jason A Mares, Anurag Sharma, Thomas G Tedesco, Juan F Idiarte, Helen Y Figueroa, Jia Guo, Karen E Duff, Tal Nuriel

**Affiliations:** ^1^ Department of Cell Biology and Pathology, New York, NY, USA; ^2^ Columbia University Irving Medical Center, New York, NY, USA; ^3^ Taub Institute for Research on Alzheimer's Disease and the Aging Brain, New York, NY, USA; ^4^ Zuckerman Institute, Columbia University, New York, NY, USA; ^5^ University College London, Long, United Kingdom; ^6^ UK Dementia Research Institute, London, United Kingdom

## Abstract

**Background:**

Possession of the apolipoprotein E e4 (*APOE4*) allele remains the most significant genetic risk factor for developing the sporadic form of Alzheimer's disease (AD). Recent attempts to characterize *APOE4*'s role in AD have suggested that carrying the *APOE4* allele may directly modulate the global pathogenesis of intracellular tau tangles. However, it remains uncertain if possession of *APOE4* can alter this in a localized manner more consistent with the pathology observed in AD.

**Methods:**

To recapitulate the tau propagation observed early in AD, we developed a novel humanized *APOE* mouse model that overexpresses misfolded human tau protein, localizing primarily to the entorhinal cortex (EC). Our experimental framework introduced *APOE* into a previously studied tau propagation model by crossing homozygous humanized *APOE* (*APOE3/3* and *APOE4/4*) mice to EC‐Tau mice that harbor the human *MAPT* P301L mutation under the control of a neuropsin‐tTa promoter. Male and female mice were analyzed at three‐time points (5‐7, 15‐17, and 25‐27 months) using various in‐vivo and ex‐vivo techniques to observe and measure tau pathology, neurodegeneration, and spatial memory defects.

**Results:**

We observed differential tau spread and neurodegeneration altered by *APOE* genotype in both a sex‐dependent and age‐dependent manner. Interestingly, our results point mainly to decreased tau pathology in regions associated with early AD in homozygous *APOE4* mice. Upon analysis of both the 15‐17 and 25‐27 month cohorts, we have discerned a significant decrease in tau propagation and tau‐associated cortical atrophy to the hippocampus and EC in the female *APOE4* mice. Our age‐dependent findings on neurodegeneration and tau burden were significantly modulated by sex, with male mice demonstrating an attenuated phenotype compared to their female counterparts. Additional spatial transcriptomics analysis revealed significant alterations in genes associated with neuronal and glia homeostasis in the EC and hippocampus of *APOE4* mice in both sexes.

**Conclusions:**

Here, we report on the generation and characterization of a novel *APOE*‐tau mouse model that replicates the region‐specific tau propagation observed in early AD. Our results offer novel insights into *APOE4*'s role in AD by revealing how *APOE* genotype, sex, and age alter the propagation of pathological tau along anatomically connected, vulnerable AD brain regions.

